# Size-regulated group separation of CoFe_2_O_4_ nanoparticles using centrifuge and their magnetic resonance contrast properties

**DOI:** 10.1186/1556-276X-8-376

**Published:** 2013-09-03

**Authors:** Jongeun Kang, Hyunseung Lee, Young-Nam Kim, Areum Yeom, Heejeong Jeong, Yong Taik Lim, Kwan Soo Hong

**Affiliations:** 1 Center for MR Research, Korea Basic Science Institute, Cheongwon 363-883, South Korea; 2 Graduate School of Analytical Science and Technology, Chungnam National University, Daejeon 305-764, South Korea

**Keywords:** Magnetic nanoparticles, Magnetic resonance imaging, Relaxivity, Particle size regulation

## Abstract

Magnetic nanoparticle (MNP)-based magnetic resonance imaging (MRI) contrast agents (CAs) have been the subject of extensive research over recent decades. The particle size of MNPs varies widely and is known to influence their physicochemical and pharmacokinetic properties. There are two commonly used methods for synthesizing MNPs, organometallic and aqueous solution coprecipitation. The former has the advantage of being able to control the particle size more effectively; however, the resulting particles require a hydrophilic coating in order to be rendered water soluble. The MNPs produced using the latter method are intrinsically water soluble, but they have a relatively wide particle size distribution. Size-controlled water-soluble MNPs have great potential as MRI CAs and in cell sorting and labeling applications. In the present study, we synthesized CoFe_2_O_4_ MNPs using an aqueous solution coprecipitation method. The MNPs were subsequently separated into four groups depending on size, by the use of centrifugation at different speeds. The crystal shapes and size distributions of the particles in the four groups were measured and confirmed by transmission electron microscopy and dynamic light scattering. Using X-ray diffraction analysis, the MNPs were found to have an inverse spinel structure. Four MNP groups with well-selected semi-Gaussian-like diameter distributions were obtained, with measured T_2_ relaxivities (*r*_2_) at 4.7 T and room temperature in the range of 60 to 300 mM^−1^s^−1^, depending on the particle size. This size regulation method has great promise for applications that require homogeneous-sized MNPs made by an aqueous solution coprecipitation method. Any group of the CoFe_2_O_4_ MNPs could be used as initial base cores of MRI T_2_ CAs, with almost unique T_2_ relaxivity owing to size regulation. The methodology reported here opens up many possibilities for biosensing applications and disease diagnosis.

**PACS:**

75.75.Fk, 78.67.Bf, 61.46.Df

## Background

Magnetic resonance imaging (MRI) is a powerful diagnostic modality for noninvasive *in vivo* imaging due to its high resolution, lack of exposure to radiation, superior soft tissue contrast, and large image window. However, it has less sensitivity than nuclear medicine and fluorescence imaging when monitoring small tissue lesions and molecular or cellular activities [1]. Contrast agents (CAs) can improve the contrast and specificity in particular target regions of MR images, and these are widely used to produce brighter and darker areas with T_1_ and T_2_ CAs, respectively. T_2_ CAs, mainly based on iron oxide magnetic nanoparticles (MNPs), provide dark contrast in T_2_- or T_2_*-weighted (T_2_*-W) MR images depending on the T_2_ relaxivity of *r*_2_ and the MNP concentration in the region of interest [2]. Superparamagnetic iron oxide (SPIO) nanoparticles with diameters of 50 to 150 nm are thus the most commonly used MNPs in a variety of biomedical applications such as MRI contrast agents, induction of local hyperthermia, manipulation of cell membranes, biosensors, cell labeling and tracking, and drug targeting and delivery [3-8].

SPIO particles have different physicochemical and biological properties, depending on the particle size and coating material, including MR T_2_ relaxivity *r*_2_[9], cell labeling efficiency [10], cell cytotoxicity [11], and *in vivo* pharmacokinetics such as blood half-life and biodistribution [12]. Therefore, strategies by which uniform-sized biocompatible MNPs with long circulation times can be produced are highly sought after for nanomedical applications.

There are two commonly used methods for synthesizing MNPs, organometallic [13] and aqueous solution coprecipitation [14]. In the organometallic approach, the particle size can be easily controlled [15]; however, the MNPs are only soluble in nonpolar and moderately polar organic solvents. This brings about the requirement for hydrophilic and biocompatible polymer coating to make them soluble enough for *in vivo* uses [16-18]. On the other hand, the aqueous solution coprecipitation method results in nanoparticles that are intrinsically water-soluble; however, the particle size distribution is relatively wide, resulting in nonuniform contrast in T_2_- or T_2_*-W MR images. Size-controlled water-soluble nanoparticles provide the possibility to achieve uniform functionalization of their surfaces with other imaging probes such as fluorescent dyes and radiolabeled probes or with targeting molecules such as antibodies, peptides, and genes, as well as therapeutics [18,19]. Several reports are available regarding the size regulation of MNPs synthesized by coprecipitation, including a temperature-controlled coprecipitation method that requires specialized equipment and a piezoelectric nozzle method [20,21]. These processes are either highly complex or relatively ineffective owing to the requirement for a high level of control over parameters such as temperature during the synthesis. In addition, the produced particles still have an inadequate size distribution. The piezoelectric nozzle method is more effective for controlling the size; however, this technique requires specialized equipment such as a piezoelectric transducer and a frequency amplifier.

To address these issues, a facile method for controlling the MNP core size via the coprecipitation process is introduced here. Initially, we synthesized CoFe_2_O_4_ nanoparticles using an aqueous solution coprecipitation method and then separated the particles into four groups depending on their size by employing a variety of centrifugation speeds. The physicochemical properties of the four groups were subsequently evaluated. The size distribution was assessed by transmission electron microscopy (TEM) and dynamic light scattering (DLS), crystallographic confirmation was carried out by X-ray diffraction (XRD), the water proton T_2_ relaxation rate (*R*_2_) versus Co/Fe concentration was evaluated, and MR image contrast was measured at 4.7 T.

## Methods

### Synthesis of CoFe_2_O_4_ nanoparticles

The CoFe_2_O_4_ MNPs were synthesized by an aqueous solution coprecipitation method reported previously [14]. Initially, the reagents, 0.5 M FeCl_3_·6H_2_O (≥98%; Sigma-Aldrich, Tokyo, Japan) and 0.25 M CoCl_2_·6H_2_O (99% to 102%; Sigma-Aldrich), were mixed in an aqueous solution, giving a Co/Fe ratio of 1:2. The reaction mixture was stirred vigorously for 6 h in boiling distilled water with 1 M NaOH (96%; Junsei, Tokyo, Japan), and then, the resulting dark brown suspension was centrifuged at 1,771 × *g*. The precipitate was dissolved in a 2-M HNO_3_ solution with stirring for 20 min and then centrifuged again at 1,771 × *g*. The resulting precipitate was dissolved in 0.5 M Fe(NO_3_)_3_ (≥98%; Sigma-Aldrich) and stirred vigorously for 30 min at 100°C. After the reaction, centrifugation at 1,771 × *g* and redispersion in distilled water were performed three times. Finally, the suspension was dissolved in water and stored at room temperature until further use.

### Size selection of MNPs and synthesis of SiO_2_-coated MNPs

As the synthesized MNPs had a broad size distribution between 5 and 300 nm, they were separated depending on their size by stepwise centrifugation. A high-speed vacuum centrifuge system was used (SUPRA 25K; Hanil Scimed, Gangneung, Korea), with five different speeds of 1,771 × *g*, 2,767 × *g*, 11,068 × *g*, 24,903 × *g*, and 35,860 × *g* in order to separate the synthesized particles into four groups. Firstly, aggregated particles were removed by down-sinking with 1,771 × *g* for 1 h. The remaining mixture was centrifuged at 35,860 × *g* for 1 h, and then, the suspended solution was removed. Resuspension of the bottom layer provided the initial MNP solution. This was then centrifuged at 2,767 × *g*, 11,068 × *g*, and 24,903 × *g* for 1 h, with the bottom layer collected as groups A, B, and C, respectively. The first suspended solution remaining after centrifugation at 24,903 × *g* was labeled as group D. The MNPs of group C were selected for SiO_2_ coating for further applications. SiO_2_ coating was done as follows: the MNPs of group C were stabilized with polyvinylpyrrolidone (PVP) to disperse them homogeneously, and then, tetraethoxysilane solution was polymerized on the surface of PVP-stabilized CoF_2_O_4_ MNPs by adding ammonia solution as a catalyst to form SiO_2_ coating on the MNPs. The volume ratio of the ammonia solution was 4.2% to control the SiO_2_ shell thickness of the final SiO_2_-coated MNPs in this process.

### MNP characterization

The crystal shapes and structures of the synthesized MNPs in each group, in addition to the SiO_2_-coated MNPs, were measured and confirmed by TEM (Tecnai G2 F30, FEI, Hillsboro, OR, USA) and XRD (XPERT MPD, Philips, Amsterdam, The Netherlands). The XRD patterns were compared with a typical XRD spectrum of a CoFe_2_O_4_ crystal. The hydrodynamic diameter distribution of the particles was measured by DLS (UPA-150l, Microtrac, Montgomeryville, PA, USA), and the size distribution was verified from the TEM images.

In order to compare T_2_ relaxivities (*r*_2_) of the four groups and the SiO_2_-coated MNPs, the T_2_ relaxation times were measured against the Co/Fe concentration in a range below 1 mM Fe using a spin-echo pulse sequence (multi-spin multi-echo) on a 4.7-T animal MRI system (Biospec 47/40; Bruker, Karlsruhe, Germany). The amount of Co/Fe in each group was measured using an inductively coupled plasma atomic emission spectrometry system (Optima 4300DV, PerkinElmer, Waltham, MA, USA). For the MRI experiment, the MNPs were sampled at four different Co/Fe concentrations of 1.0, 0.75, 0.5, and 0.25 mM Co/Fe in distilled water in 250-μl microtubes. The MRI parameters used were as follows: TE/TR = 10/10,000 ms, number of scans = 2, slice thickness = 1 mm, FOV = 5 × 5 cm^2^, number of slices = 1. T_2_ contrast differences depending on Fe concentration for the separated groups were also compared in T_2_-W MR images.

## Results and discussion

**Figure 1:**
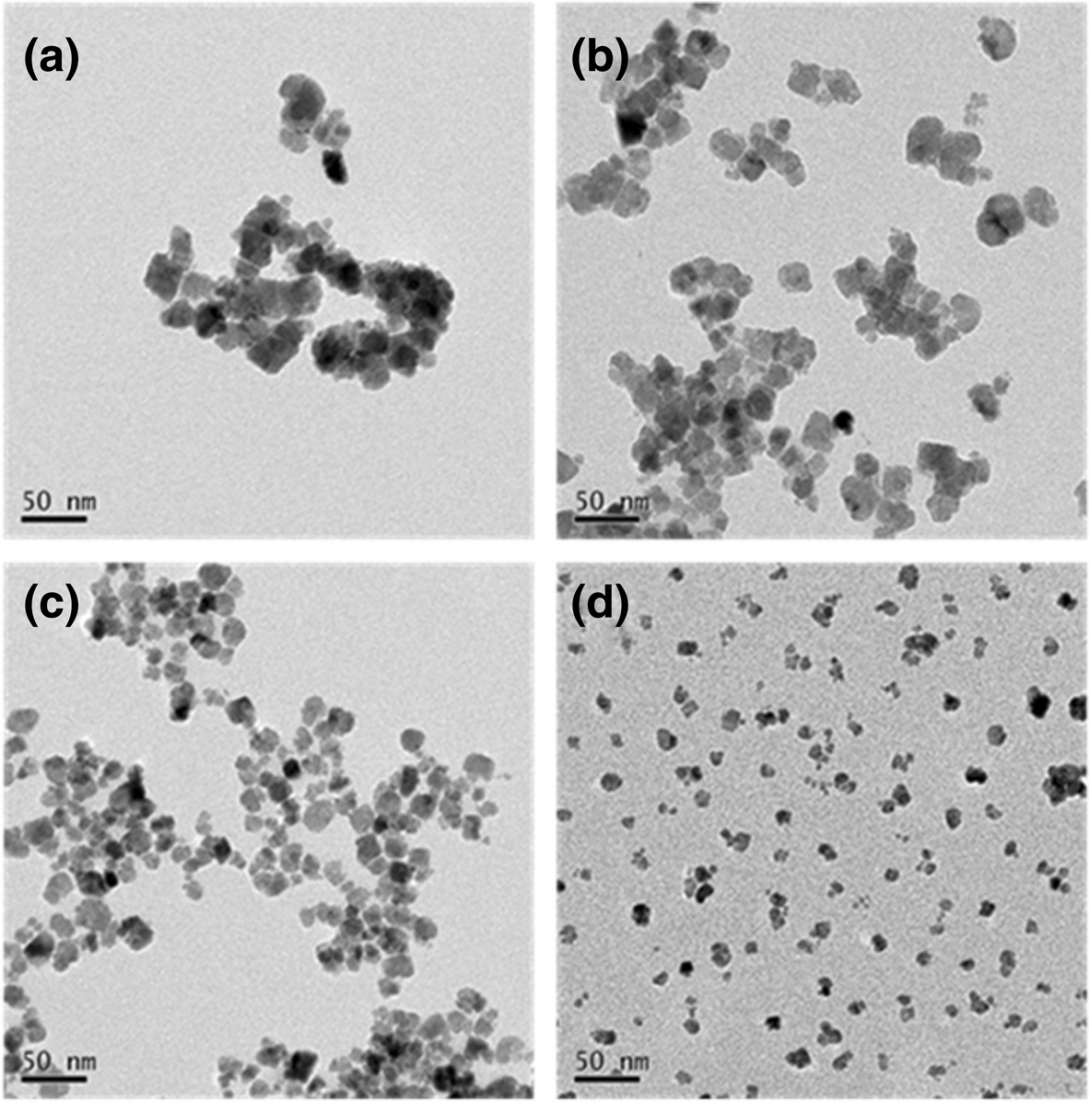
**TEM images of the four MNP groups.** The TEM images show that the particles were well dispersed and size-regulated according to the group. The mean diameters for the four groups were 24.2 ± 3.6, 20.0 ± 3.6, 15.8 ± 3.6, and 10.5 ± 2.4 nm, for groups **a** to **d**, respectively.

**Figure 2:**
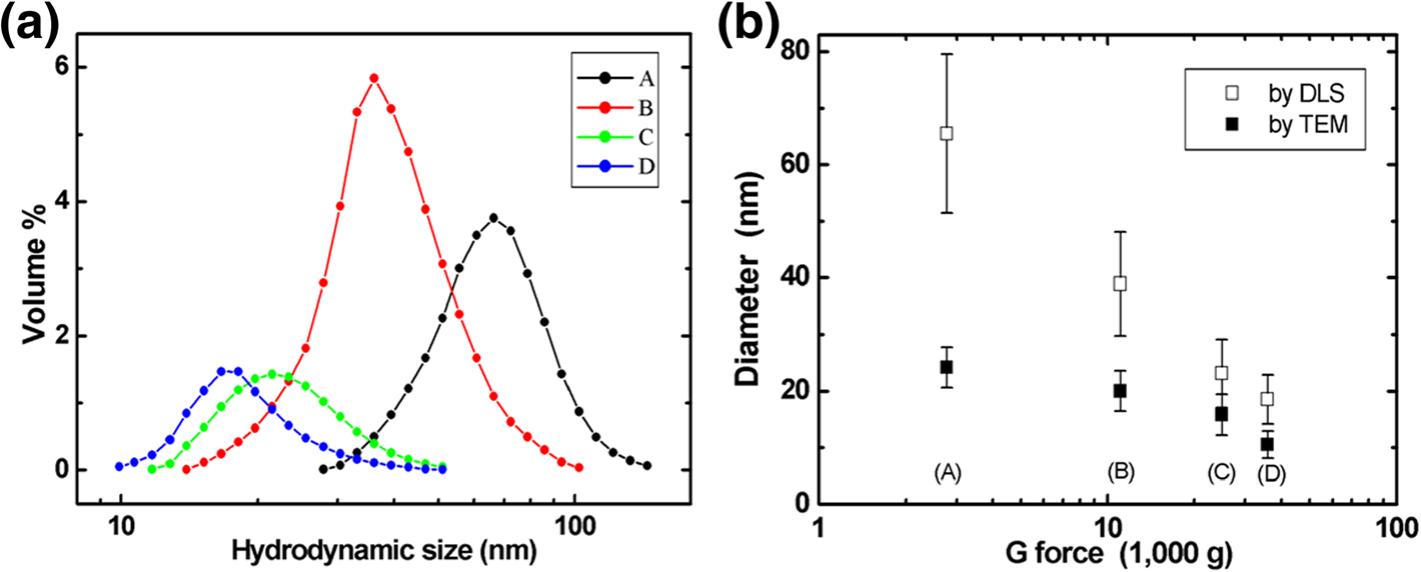
**Relative size distributions of separated MNP groups and correlation between DLS and TEM results.** Relative size distributions of separated MNP groups in aqueous solution measured by DLS **(a)** and a graph showing correlation between DLS and TEM results **(b)**. The mean DLS diameters for the four groups, A to D, were 65.5 ± 14.0, 38.9 ± 9.1, 23.1 ± 6.0, and 18.5 ± 4.4 nm, respectively, with relative volumes of 29% (A), 49% (B), 12% (C), and 10% (D) as measured by integration of the DLS spectra.

The MNPs synthesized by the coprecipitation method were found to have an extremely broad size distribution [14]. This characteristic would likely result in nonuniform contrast in MR images. The purpose of the present study was to overcome this limitation by separating the different sizes of particles by centrifugation. After the initial removal of aggregates, the nanoparticles were sequentially centrifuged at speeds 2,767 × *g*, 11,068 × *g*, 24,903 × *g*, and 35,860 × *g*, producing groups A, B, C, and D, respectively. As shown in the TEM images in Figure 1, the centrifugation process resulted in four groups containing particles relatively uniform in size. The mean diameters measured from approximately 100 randomly selected particles from each group were found to be 24.2 ± 3.6, 20.0 ± 3.6, 15.8 ± 3.6, and 10.5 ± 2.4 nm for groups A, B, C, and D, respectively. As the rotational speed increased, the MNP diameters decreased, with significant differences between adjacent groups (*P* < 0.01). The hydrodynamic diameter distributions of the MNPs in the four groups were Gaussian-like, with values of 65.5 ± 14.0, 38.9 ± 9.1, 23.1 ± 6.0, and 18.5 ± 4.4 nm (Figure 2a) and volume ratios of 29%, 48%, 13%, and 10% for groups A to D, respectively. Further, from the measured volume ratios in Figure 2a, the highest MNP volume was observed for group B; groups C and D could also provide an adequate quantity of uniform-sized MNPs for use in applications that require very small sized (approximately 10 nm) MNPs. The amount of synthesized MNPs from group D was approximately 0.5 g, which could be easily scaled-up using a larger reaction vessel.

The mean diameter of the MNPs, as measured by TEM and DLS, decreased as the centrifugation speed decreased (Figure 2b), indicating that the MNP particles synthesized by the coprecipitation method were well separated and clearly resolved into the four groups by the different centrifugation speeds.

**Figure 3:**
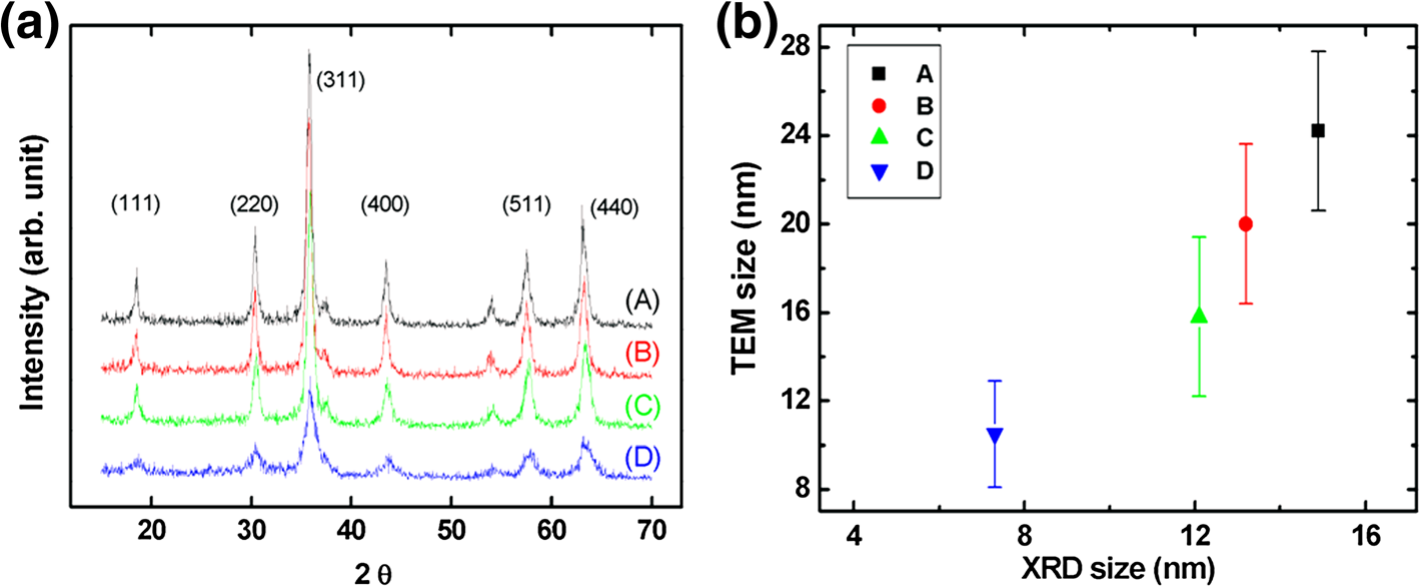
**A stack plot of XRD patterns of MNPs and size calculation.** The nanoparticles were well crystallized, and the peaks are in accordance with the typical CoFe_2_O_4_ XRD spectrum in which the main peaks are (111), (220), (311), (400), (511), and (440) **(a)**. The mean diameters of the crystal particles calibrated from signal width for the four groups from A to D were 14.9, 13.2, 12.1, and 7.3 nm, respectively **(b)**.

Using the organometallic method reported by others, the particle size of MNPs can be easily controlled, with a narrower diameter distribution achievable in comparison to the combined coprecipitation and centrifugation methods described here. However, the amount of MNPs that can be synthesized in a single process is quite small, and these have the added disadvantage of being hydrophobic. A coating is therefore necessary in order to render these MNPs hydrophilic and to enable them to be used for functions such as drug loading, targeting, or imaging probes (PET or fluorescence). Even though the size distribution of MNPs synthesized by the coprecipitation method was large, huge amounts of size-controlled MNPs were obtained by combining the method with a simple centrifugation process. Figure 3a shows the XRD results obtained for the four groups of CoFe_2_O_4_ MNPs. All groups can be seen to exhibit the same peaks, which match well with the standard Fe_3_O_4_ XRD pattern (JCPDS 75–0030). The mean particle size (*D*) can be calculated by the full-width at half-maximum (FWHM) and the area/height ratio (*β*) of the XRD peaks with instrumental correction, using the equation *D* = *Kλ* / *β* × cos*θ*, where *K* is the Scherrer constant, *λ* is the wavelength, *β* is the FWHM (in radians), and *θ* is the peak angular position [22,23]. The XRD information gave crystallite sizes of 14.9, 13.2, 12.1, and 7.3 nm (Figure 3). As MNPs synthesized by coprecipitation may contain some iron oxide crystals, the particle size calculated from the TEM images was larger than that from the XRD data (Figure 3b).

**Figure 4:**
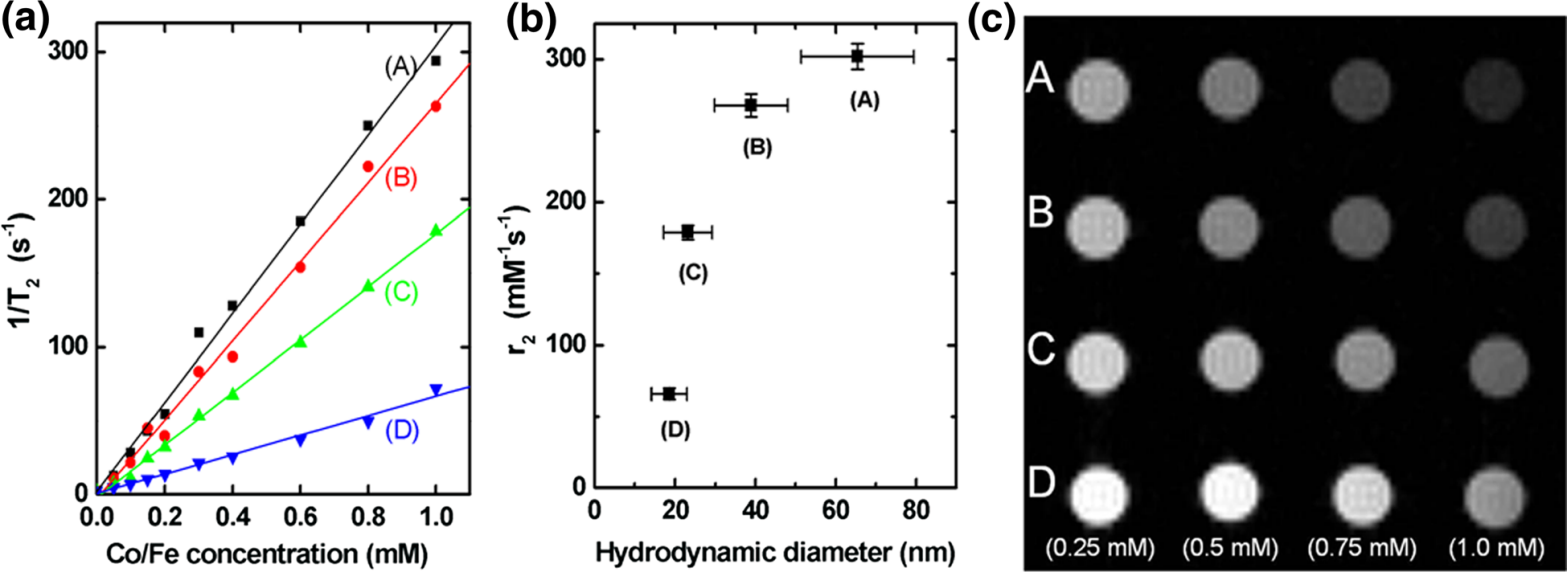
**Calculated T**_**2**_**relaxation rates and relaxivity and representative MR image for the four groups.** Concentration-dependent T_2_ relaxation rates (1/T_2_) **(a)**, calculated T_2_ relaxivity *r*_2_**(b)** for the four groups at 4.7 T (200 MHz for protons), and representative MR image **(c)** for the four groups depending on the Co/Fe concentration. The slopes of the fitted lines provide the T_2_ relaxivity (*r*_2_) at the concentration of 1 mM for each group; the values are 302 ± 9, 268 ± 8, 179 ± 5, and 66 ± 4 mM^−1^s^−1^ for groups A, B, C, and D, respectively. A representative T_2_-weighted MR image (TE/TR = 10/10,000 ms, slice thickness = 2 mm, number of scans = 2), obtained by a conventional spin-echo pulse sequence on a 4.7-T MRI system, from the samples with four different Co/Fe concentrations (0.25, 0.5, 0.75, and 1.0 mM) for the groups A to D is shown **(c)**. The signal decrease due to T_2_ negative contrast is higher with increasing particle size and increasing Co/Fe concentration, especially for group A, which is in accordance with the result shown in **(a)**.

**Figure 5:**
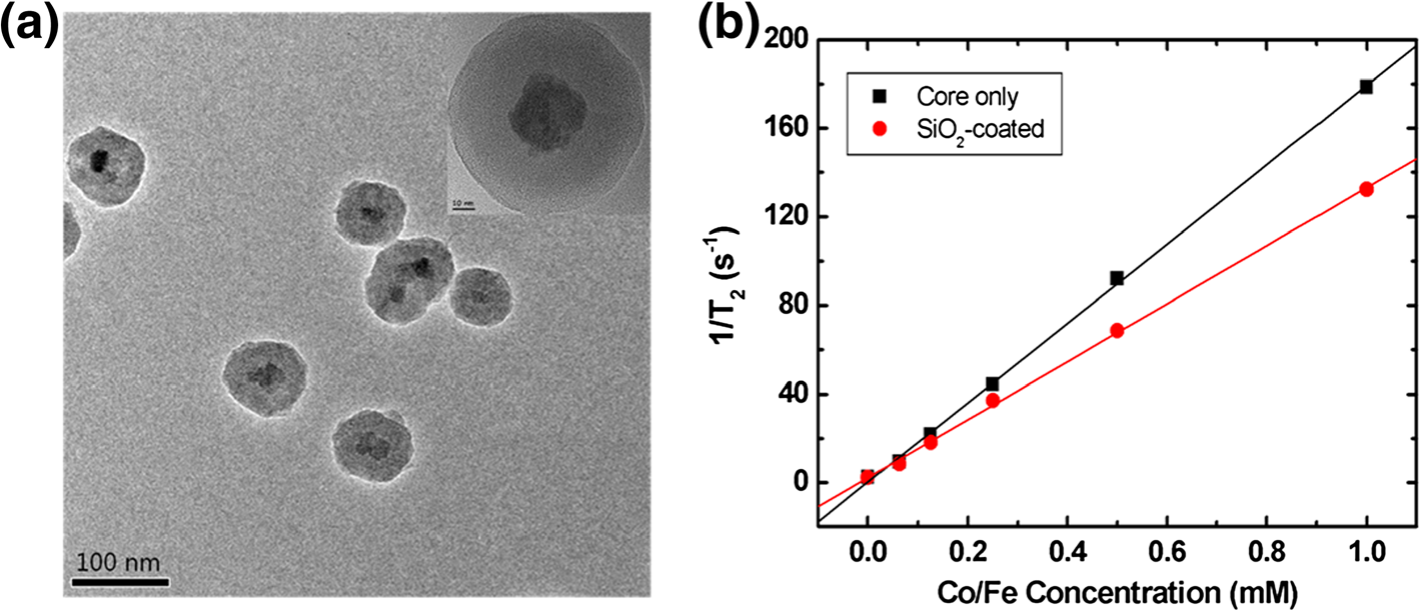
**TEM images (a) and T**_**2**_**property measurement (b) of the SiO**_**2**_-**coated MNPs.** The TEM images show that the particles consisted of core CoFe_2_O_4_ nanoparticles and a SiO_2_ coating with a shell thickness of approximately 25 nm, providing a total particle diameter of 70.8 ± 4.3 nm (note the inset for the particle shape in detail). The measured *r*_2_ was 130 ± 2 mM^−1^s^−1^, which was 27% smaller than that of the MNP group C core alone.

The size-dependent MR contrast (T_2_ relaxivity) of the MNPs was measured on a 4.7-T MRI system. Figure 4a shows the dependence of the T_2_ relaxation rate (*R*_2_, s^−1^) on the MNPs of the four groups. The T_2_ relaxation rate was increased with increased Co/Fe concentration, and the T_2_ relaxivities (*r*_2_) for the groups were measured from the slopes of the data. The *r*_2_ values were found to be 302 ± 9, 268 ± 8, 179 ± 5, and 66 ± 4 mM^−1^s^−1^ for groups A, B, C, and D, respectively (Figure 4b). These values are comparable to those in the study of Joshi et al. [24], in which the T_2_ relaxivity of cobalt ferrite nanostructures synthesized by the thermal decomposition method was reported to be 110 to 301 mM^−1^s^−1^ depending on the particle size (6 to 15 nm). Figure 4c shows an MRI phantom image with the four groups depending on the Co/Fe concentration measured on the 4.7-T MRI system. The increase in MR T_2_ negative contrast was shown to depend on both the particle diameter and the Co/Fe concentration, indicating that a well-controlled contrast with each size-selected group of MNPs could be obtained. The particle size dependence of T_2_ relaxivity was in accordance with other reports [25,26], in which T_2_ spin-spin relaxation is affected by mass magnetization depending on the magnetic particle size in the range lower than approximately 1 μm. This demonstrates that each group of MNPs could be used for specific applications depending on the particle diameter. One concern regarding these as-prepared MNPs is that they are not stable to variations in pH. This is a problem that needs to be overcome if they are to be successfully employed *in vivo*. We therefore investigated the coating of the MNPs with a stable and biocompatible material such as SiO_2_ to enhance stability and avoid potential toxic effects on cells (Figure 5) [19]. The T_2_ relaxivity of the SiO_2_-coated MNPs made from group C was 130 ± 2 mM^−1^s^−1^ (Figure 5b), which was approximately 27% lower than that of the original core particles. Group C was selected for SiO_2_ coating in order to get final SiO_2_-coated SPIO MNPs with a diameter of 50 to 100 nm and with a moderate T_2_ relaxivity value. The SiO_2_ coating would facilitate the addition of therapeutic and targeting functions such as drugs and antibodies to the MNPs, enabling them to serve as both imaging agents and a therapeutic carrier species.

There have been several reports on Fe_3_O_4_-based MNPs with a narrow size distribution made by the coprecipitation method. Lee et al. used a piezoelectric nozzle [20], which, despite effectively controlling the particle size, requires specialized equipment and many steps. Jiang et al. employed a coprecipitation methodology using urea, which provided SPIO MNPs with a narrow size distribution [27]. The average diameter of these MNPs could be adjusted from 8 to 50 nm depending on the decomposition of urea in the ferrite solution; however, they required additional dextran coating in order to make them water soluble. In the present study, the use of centrifugation in combination with the coprecipitation method enabled effective regulation of the size of the MNPs without the requirement for a specialist. A large quantity of each size of particles could be produced, overcoming many of the shortcomings of the coprecipitation method.

## Conclusions

A simple centrifugation technique was combined with a coprecipitation method in aqueous solution in order to obtain four groups of CoFe_2_O_4_ MNPs. These were successfully produced in large quantities, with different diameters and MRI T_2_ relaxivity values and narrow size distributions, depending on the centrifugation speed. The obtained MNPs had a strong size-dependent MRI T_2_ contrast with T_2_ relaxivities between 302 and 66 mM^−1^s^−1^, providing a selection of particles from which the most appropriate for a specific application could be chosen. In the present study, the particles of group C were selected for additional SiO_2_ coating. This was to demonstrate the potential of these MNPs to be used for *in vivo* applications where they would require a long blood half-life, in addition to biocompatibility. Each of the groups of CoFe_2_O_4_ MNPs could be used as the initial base cores of MRI T_2_ contrast agents, with almost unique T_2_ relaxivity due to the size regulation. This opens up many possibilities for biosensing applications and disease diagnosis.

## Abbreviations

CAs: Contrast agents; DLS: Dynamic light scattering; FWHM: Full-width at half-maximum; MNP: Magnetic nanoparticle; MRI: Magnetic resonance imaging; SPIO: Superparamagnetic iron oxide; TEM: Transmission electron microscopy; XRD: X-ray diffraction.

## Competing interests

The authors declare that they have no competing interests.

## Authors’ contributions

JK, YTL, and KSH designed the experiments. JK, HL, AY, and Y-NK performed the experiments. JK, Y-NK, and HJ analyzed the data. JK, HL, AY, and HJ made the figures. JK and KSH wrote the manuscript. All authors read and approved the final manuscript.
